# Subinhibitory Arsenite Concentrations Lead to Population Dispersal in *Thiomonas* sp.

**DOI:** 10.1371/journal.pone.0023181

**Published:** 2011-08-18

**Authors:** Marie Marchal, Romain Briandet, David Halter, Sandrine Koechler, Michael S. DuBow, Marie-Claire Lett, Philippe N. Bertin

**Affiliations:** 1 Génétique Moléculaire, Génomique et Microbiologie, UMR7156 CNRS & Université de Strasbourg, Strasbourg, France; 2 INRA, UMR1319 MICALIS, Jouy-en-Josas, France; 3 AgroParisTech, UMR MICALIS, Massy, France; 4 Université Paris-Sud 11, Institut de Génétique et Microbiologie, CNRS UMR 8621, Orsay, France; University of British Columbia, Canada

## Abstract

Biofilms represent the most common microbial lifestyle, allowing the survival of microbial populations exposed to harsh environmental conditions. Here, we show that the biofilm development of a bacterial species belonging to the *Thiomonas* genus, frequently found in arsenic polluted sites and playing a key role in arsenic natural remediation, is markedly modified when exposed to subinhibitory doses of this toxic element. Indeed, arsenite [As(III)] exposure led to a considerable impact on biofilm maturation by strongly increasing the extracellular matrix synthesis and by promoting significant cell death and lysis within microcolonies. These events were followed by the development of complex 3D-biofilm structures and subsequently by the dispersal of remobilized cells observed inside the previously formed hollow voids. Our results demonstrate that this biofilm community responds to arsenite stress in a multimodal way, enhancing both survival and dispersal. Addressing this complex bacterial response to As(III) stress, which might be used by other microorganisms under various adverse conditions, may be essential to understand how *Thiomonas* strains persist in extreme environments.

## Introduction

To cope with environmental stresses, microorganisms have evolved multiple survival strategies, including the synthesis of detoxification enzymes and the acquisition of resistance genes by horizontal gene transfer. These defence mechanisms may also rely on multicellular behaviours, such as those observed inside biofilms, where a part of the bacterial population can die to promote the survival of the remaining cells [1]. Biofilms, which are surface-attached microbial communities embedded in an extracellular matrix, exhibit significantly higher survival capacities in extreme conditions compared to their planktonic counterparts [2]. They constitute the most common lifestyle of microbial communities, providing them with several advantages. For example, the extracellular matrix allows the development of a structured community with subsequent physiology and genetic diversity [3]. To date however, little is known regarding the behaviour of environmental bacterial biofilms despite their roles in numerous processes, either deleterious, *e.g.* biocorrosion and remobilisation of toxic elements, or beneficial, *e.g.* bioremediation [4], [5]. Due to the significance of these processes, their understanding and the prediction of their evolution are of prime importance and require a better knowledge of the mechanisms governing biofilm formation and persistence.

Members of the *Thiomonas* genus are ubiquitous in extreme environments contaminated by arsenic [6], [7], one of the most toxic inorganic elements currently known [8]. The acid mine drainage (AMD) waters of the former mine of Carnoulès, Gard (France), contain high arsenic levels (up to 4.66 mM) mainly in the form of arsenite As(III). Interestingly, this site is subjected to a natural process of remediation in which *Thiomonas* bacterial strains play a key role by oxidizing As(III) into As(V) which co-precipitates with iron and sulfur [7], [9]. These Betaproteobacteria, defined as facultative chemolithoautotrophs, which grow optimally in mixotrophic media containing reduced inorganic sulfur compounds (RISCs) and organic supplements, are also of particular interest for their use in bioremediation [10]. Indeed, they have developed several resistance and adaptive mechanisms to cope with arsenic stress, mainly by the means of detoxification enzymes. In addition, these strains have been shown to possess the ability to form biofilms [11], [12]. However, little is known regarding the factors influencing the development of these structures and how these organizations may play a role in the persistence of strains in such extreme environments.

With this aim in view, we explored the effects of subinhibitory As(III) concentrations on both biofilm formation and development in *Thiomonas* sp. CB2. This strain, previously isolated from the Carnoulès AMD waters, is not capable to grow in the absence of any inorganic energy source, *i.e.* reduced inorganic sulfur compounds (RISCs) or As(III), and its genome carries an operon *aox* coding for the arsenite oxidase enzyme. Interestingly, *Thiomonas* sp. CB2 has been shown to form flocs when cultivated under dynamic conditions and to form important biofilms under static growth conditions [11]. In the present study, using multimodal confocal laser scanning microscopy, we show that the structural dynamics of the biofilm is markedly changed when bacteria are exposed to As(III). Importantly, our results highlight how this process may allow the strain to survive and also develop in such toxic ecosystems.

## Materials and Methods

### Bacterial strain and culture conditions

*Thiomonas* sp. CB2 was previously isolated from the acid mine drainage waters of Carnoulès, Gard (France) [11]. The strain was cultivated on m126 (modified 126 medium) liquid or solid medium as previously described [12]. Arsenic was added at the desired concentration from sterile stocks of 667 mM of the metalloid ion in deionized water, from either NaAsO_2_ [As(III)] or Na_2_HAsO_4_•7H_2_O [As(V)] salts (Prolabo, Fontenay-sous-bois, France). Planktonic growth was assessed in liquid medium in the absence and in the presence of either 1.33 or 2.67 mM As(III) as follows: an exponential culture was diluted to an OD_600_ of 0.001 on a Spectronic Genesis spectrophotometer and growth was then assessed by measuring daily OD_600_ of the cultures. The minimum inhibitory concentration (MIC) of As(III) for this strain has been previously determined to be 10 mM as follows: cultures were performed in triplicate on gelified medium supplemented with 10, 5, 2.25, 1.25 or 0 mM As(III) and were incubated at 30°C for up to 10 days. MIC was defined as the minimal As(III) concentration tested inhibiting growth [11], [12].

Biofilm cultures were performed in microscopic quality 35-mm polystyrene dishes (ibiTreat low 35-mm μ-Dish, Integrated BioDiagnostics, Martinsried, Germany) as follows: 1 mL of m126 medium, supplemented or not with As(III), was inoculated with 10 µL of an exponentially growing culture. Incubation was performed at 30°C under stagnant culture conditions. The medium was renewed every day to avoid growth limitation due to nutrient depletion.

### Confocal Laser Scanning Microscopy

Biofilms were grown in microscopic quality 35-mm polystyrene dishes as described above. Before staining, samples were gently rinsed with 1 mL of 150 mM NaCl to remove loosely adherent planktonic cells. Cell viability was assessed by probing the cells for 15 min with 50 µL of a stock solution of SYTO9 (6 µM), a permeable nucleic acid fluorescent probe dye, and SYTOX Red (5 nM), a cell impermeable probe (Molecular Probes, Invitrogen Corporation, Cergy Pontoise, France) [13]. This combination was chosen for the spectral separation of these dyes, as previously demonstrated for equivalent stains [14]. Polysaccharides within the exopolymeric matrix of the biofilm were stained with 50 µL of Alexa Fluor 633 conjugated to Concanavalin A (ConA) or Wheat Germ Agglutinin (1 mg mL^−1^) (Molecular Probes, Invitrogen Corporation).

*Z*-stacks of horizontal plane images of biofilm were acquired with a step of 1 µm using multimodal confocal scanning laser microscopy (Leica TCS SP2 AOBS, Leica Microsystems, Nanterre, France) with a ×63 (1.4 N.A.) oil immersion lens. Images were recorded at an excitation wavelength of 488 nm and emission wavelengths from 500 to 530 nm for SYTO9 and at an excitation wavelength of 633 nm and emission wavelengths from 657 to 757 nm for SYTOX Red and Alexa Fluor 633 conjugated to Concanavalin A or Wheat Germ Agglutinin. The surface coverage was quantified using NIH ImageJ analysis software (http://rsbweb.nih.gov/ij/). A manual threshold was applied to binarize images. Simulated 3D fluorescence projections were generated using IMARIS 7.0.0 software (Bitplane, Zürich, Switzerland). Dead (SYTOX Red), total cells (SYTO9) and exopolysaccharide (ConA) biovolumes were quantified from images series using PHLIP, a freely available Matlab-based image analysis toolbox (www.phlip.org). The biovolume represents the overall volume of a biofilm (µm^3^) and may be used to estimate its total biomass. It has been defined as the number of foreground pixels in an image stack multiplied by the voxel volume, which is the product of the squared pixel size and the scanning step size [15]. To limit the temporal variability between biofilm samples, all replicates for confocal microscopy experiments were performed during a single week. In each condition, six stacks were randomly acquired on two independent samples from six microscopic fields of the central 35-mm dish zone.

### Nuclease treatment of biofilms

To test the role of extracellular nucleic acids on the biofilm structure, Benzonase® Nuclease (Sigma-Aldrich, St Louis, MO, USA) was added to 72 h-old biofilms to a final concentration of 125 U mL^−1^ (a control without Benzonase® Nuclease was also included). After 1 h of incubation at 37°C, biofilm supernatant was removed to eliminate non-attached cells. Biofilm integrity was determined, after staining of the attached cells with SYTO9 as described above, at a magnification of 1000× under oil immersion using a Leica DM 4000 B epifluorescence microscope equipped with a Leica DFC300 FX digital camera (Leica Microsystems). Images were recorded at an excitation wavelength of 488 nm and emission wavelengths from 500 to 550 nm.

### As(III) resistance assay

As(III) resistance of 72 h-old biofilms grown in the absence or in the presence of 1.33 mM As(III) was assessed by plating biofilm cells (washed in 150 mM NaCl) on solid m126 medium containing 10.7 mM As(III). The minimal inhibitory concentration for this strain has been previously determined to be 10 mM [11]. Total biofilm cell concentrations were obtained by plating serial dilutions of the biofilm population on solid m126 medium containing no As(III). Colony forming units (CFUs) were counted after 2 weeks of incubation at 30°C. As(III) resistance of the biofilm cell population was expressed as the percent of cells able to grow in the presence of 10.7 mM As(III).

### Identification of potential exopolysaccharide synthesis coding genes

Genes possibly involved in exopolysaccharide synthesis were searched for in *Thiomonas* sp. CB2 genome using available genomic data, *i.e.* the genome sequence of *Thiomonas* sp. 3As (http://www.cns.fr/agc/microscope/mage/index.php?) and comparative genomic hybridization (CGH) data between *Thiomonas* sp. CB2 and *Thiomonas* sp. 3As (available in the ArrayExpress database with the accession number E-MEXP-2260) [11].

### RNA extraction

Biofilms were grown as described above, in the absence or in the presence of 1.33 mM As(III). After 72 h of incubation, biofilm cells were manually recovered by scratching the dishes. Cells were then harvested by centrifugation and stored at −80°C to prevent RNA degradation. Pellets were suspended in 400 µL of suspension solution (25 mM Tris-HCl pH 7.6–10 mM EDTA and 20% glucose (vol/vol)) and transferred into microtubes containing 500 µL of acidic phenol (pH 4.5) and 0.4 g of 0.1 mm-diameter glass beads (VWR, Fontenay-sous-Bois, France). Cells were mechanically broken with a “FP120 FastPrep Cell Disruptor” (Bio 101, Q-BIOgene, Illkirch, France) (two 30 sec cycles of homogenization at maximum speed with 1 min interval on ice). Total RNA were then extracted with TRIzol Reagent (Invitrogen Corporation) according to the manufacturer's instructions. RNA aliquots were purified with the RNeasy Plus mini kit (Qiagen, Courtaboeuf, France) to ensure the elimination of genomic DNA. RNA integrity was checked by automated electrophoresis with the Experion system (Bio-Rad, Marnes-la-Coquette, France). Total RNA concentration was determined spectrophotometrically with a Nanodrop spectrophotometer (NanoDrop Technologies, Wilmington, DE, USA).

### Quantitative real time PCR

Total RNA (2 µg) for each condition was reverse transcribed using Maxima® First Strand cDNA Synthesis Kit for RT-qPCR (Fermentas, Saint-Rémy-lès-Chevreuse, France). The cDNA synthesis reaction was completed in 30 min at 50°C. Real time quantitative PCR was carried out with a MyiQ single-color Real-time PCR detection system (Bio-Rad). The reaction mixture contained 12.5 µL of iQ™SYBR®Green Supermix (Bio-Rad), 5 µL of cDNA (diluted 1/3) and 300 nM of each primer in a total volume of 25 µL. Thermocycling conditions were as follow: 5 min at 95°C and 40 cycles of 15 sec at 95°C, 15 sec at 62°C and 1 min at 72°C. For each quantitative PCR run, non-template controls were performed to identify false positives. Negative controls without reverse transcriptase were also performed for each cDNA synthesis reaction and verified in real time PCR to determine the presence of contaminating genomic DNA. Absence of primer dimers was checked. Two biological replicates (independent cultures) and two technical quantitative PCR replicates were performed for each experiment. The data were analyzed with the Relative Expression Software Tool [16]. Statistical significance was defined at *p*-value<0.05.

House-keeping genes were chosen on the basis of previous transcriptomic experiments on arsenic stress [17]. Genes with a stable expression between cultures performed with and without As(III) were identified and homologous genes were then searched for in *Thiomonas* sp. CB2 genome using *Thiomonas* sp. 3As genome and the previously performed CGH experiments [11]. Primers were designed using *Thiomonas* sp. 3As genome to generate amplification products smaller than 200 bp in size. The pairs of primers used are listed in Table S1. Three housekeeping genes, *i.e.* THI_2580 coding for a putative S-adenosyl-L-methionine-dependent methyltransferase, THI_0556 coding for a DnaA initiator-associating protein and THI_1854 coding for a putative transcriptional regulator, were used as standards to obtain a normalized ratio for tested genes in As(III) induced samples compared to non induced. The PCR efficiency of the genes of interest and internal control genes were optimized to be similar enough by adjusting the primer concentrations to 300 nM each (data not shown).

### Statistical analysis

The ANOVA test was used to compare strain growth parameters and to evaluate the disparity between biofilm replicates. The Student's two-tailed *t*-test was used to compare the surface coverage rates and also the ConA/SYTO9 and SYTOX Red/SYTO9 biovolume ratios. These ratios were calculated by averaging the data of the six microscopic fields analyzed per biofilm culture condition. To compare the ratios between each culture conditions, Student's two-tailed *t*-tests were performed using an n of 6, assuming unequal variances equality between the different conditions. A Fisher test was performed to assess the variance equality of the different parameters between each condition. Significance of all three statistical tests was set at *p*-value<0.05 (*P*<0.05).

## Results

### Increased extracellular matrix synthesis under arsenite exposure

*Thiomonas* sp. CB2 has the capacity to form flocs when cultivated under planktonic conditions. This propensity was observed to be enhanced in the presence of 1.33 mM As(III) but not in the presence of 1.33 mM As(V) (data not shown). To gain insight into the possible effects of As(III) on biofilm formation and development, the strain was cultivated under stagnant conditions in polystyrene dishes in the absence or in the presence of subinhibitory arsenite concentrations, *i.e.* 1.33 and 2.67 mM As(III). In the range of concentrations tested here, arsenite did not significantly affect *Thiomonas* sp. CB2 planktonic growth (ANOVA test, *P*>0.05, Figure S1). To avoid growth limitation due to nutrient depletion, the growth medium was renewed every day. Both biofilm formation and development were investigated using multimodal confocal scanning and a cell viability staining.

The bacterial adhesion began after about 24 hours of culture and, after 48 hours of growth, cell adhesion was not significantly influenced by the presence of arsenite (*t*-test, *P*>0.05). The percentage of bacterial surface coverage was of 11.5±5.2 in the absence of As(III) and of 15.7±4.7 and 12.9±4.2 (mean ± SD) in the presence of 1.33 and 2.67 mM of As(III). Microcolonies were present in both the presence and absence of metalloid, the most developed multicellular structures being filled with living cells, *i.e.* labelled with SYTO9 but not with SYTOX Red (Figure S2).

**Figure 1:**
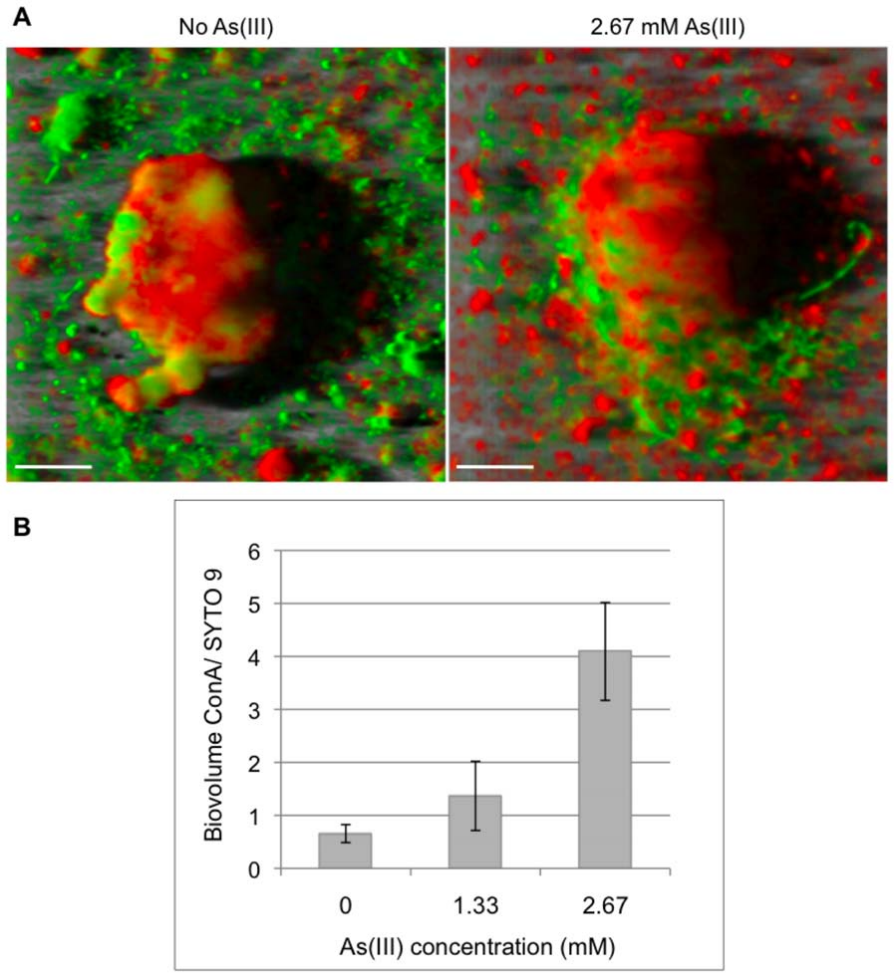
As(III)-induced exopolysaccharide biosynthesis. 72 h-old *Thiomonas* sp. CB2 biofilms were stained using SYTO9 (cells, green) and a labeled lectin, ConA (exopolysaccharides, red). **A.** Three-dimensional confocal reconstruction of representative microcolonies using IMARIS software in the absence and in the presence of 2.67 mM As(III). **B.** Biovolume ConA/SYTO9 as a function of As(III) concentration. Cells (SYTO9) and exopolysaccharide (ConA) volumes were quantified with light emission intensity using PHLIP software (www.phlip.org). For each experiment, six random microscope fields were analyzed from two replicates. An ANOVA test was performed to evaluate the disparity between replicates, indicating *P*>0.05 for each parameter. The two replicates per condition could also be considered as not significantly different. The presence of arsenite significantly influenced the biovolume ConA/SYTO9 (*t-*test, *p* = 0.047 between biofilms non-exposed to arsenite and biofilms exposed to 2.67 mM As(III)). Error bars represent standard deviations. Scale bars: 10 µm.

To get further structural information about these bacterial cell aggregates, two fluorescently labelled lectin, i.e Concanavalin A (ConA) binding selectively α-mannopyranosyl and α-glucopyranosyl residues and Wheat Germ Agglutinin (WGA) binding N-acetyl-D-glucosamine and N-acetylneuraminic acid, were used to stain the polysaccharides present within the exopolymeric matrix of the biofilm. Interestingly, after 72 hours incubation, these microcolonies were surrounded by a higher amount of extracellular polysaccharides in the presence of arsenite. These exopolysaccharides were revealed by ConA but not by WGA, which did not mark any extracellular component (data not shown). The exopolysaccharide volume revealed by ConA compared to the total cell volume revealed by SYTO9 staining was 2.1 and 6.2-fold greater in the presence of 1.33 and 2.67 mM As(III), respectively, than without As(III) (Figure 1). These data suggest that the exposure to the metalloid increases the extracellular polymer biosynthesis in *Thiomonas* sp. CB2. Genes possibly involved in the extracellular polymer synthesis were searched for using the genome of a closely-relative strain, *Thiomonas* sp. 3As, and previous CGH array data (see Material and Methods section). Four genes were identified, *i.e. rfbABCD*, which are organized in an operon-like manner in *Thiomonas* sp. 3As genome (THI_2966 to THI_2969) and code for dTDP-rhamnose biosynthesis, a potential exopolysaccharide precursor. The expression of *rfbB* and *rfbD*, THI_2969 and THI_2968, respectively, in the *Thiomonas* sp. 3As genome, was measured by quantitative RT-PCR in biofilm cells exposed or not to 1.33 mM As(III). These two genes were expressed in both conditions, although no significant variation in their expression could be detected between the two conditions tested (data not shown).

### Cell death, As(III)-exposed biofilm architecture and the role of extracellular nucleic acids in the biofilm structural integrity

**Figure 2:**
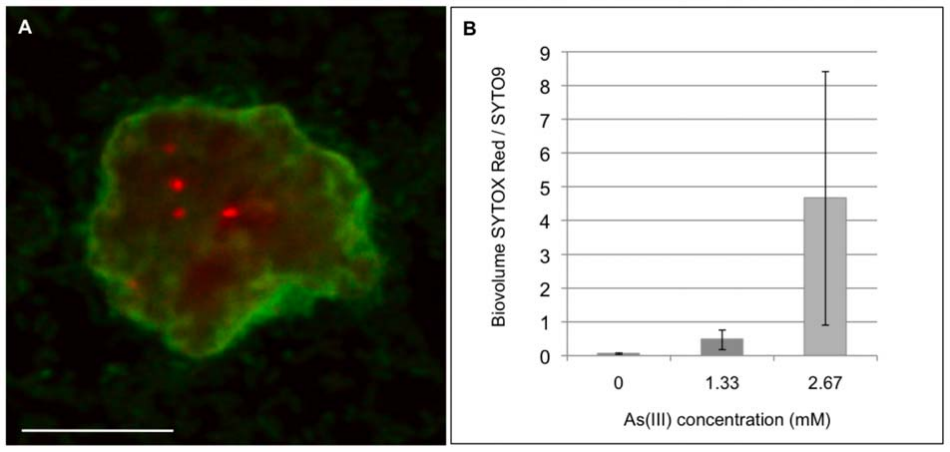
Cell death in microcolonies of 72 h-old *Thiomonas* sp. CB2 biofilms under As(III) exposure. **A.** Cell death occurs in localized regions in microcolonies. Confocal micrograph of a 1.33 mM As(III)-exposed biofilm microcolony visualised using viability stains. **B.** Biovolume SYTOX Red/SYTO9 as a function of As(III) concentration. Dead (SYTOX Red) and total cell (SYTO9) volumes were quantified using light emission intensity with PHLIP software. For each experiment, six random microscope fields from two replicates were analyzed. An ANOVA test was performed to evaluate the disparity between replicates, indicating *P*>0.05 for each parameter. The two replicates per condition could also be considered as not significantly different. The presence of arsenite presence influenced significantly the biovolume SYTOX Red/SYTO9 (*t-*test, *p* = 0.032 between biofilms non-exposed to arsenite and biofilms exposed to 1.33 mM As(III)). Error bars represent standard deviations. Scale bar: 10 µm.

When we examined the internal organization of the microcolonies within 72 h-old biofilms, we observed that they were filled with dead cells only in the presence of arsenite. The proportion of dead cells increased with arsenite concentration. The dead cell volume, compared to the total cell volume, was 8.4 and 83.2-fold greater in the presence of 1.33 and 2.67 mM As(III), respectively, than without As(III) (Figure 2). The biofilm physiological heterogeneity increased significantly with As(III) concentration (*F*-tests, *P*<0.05), variance of the SYTOX Red/SYTO9 biovolume ratios being 0.03, 0.33 and 4.05 in the absence of As(III) and in the presence of 1.33 and 2.67 mM As(III), respectively.

**Figure 3:**
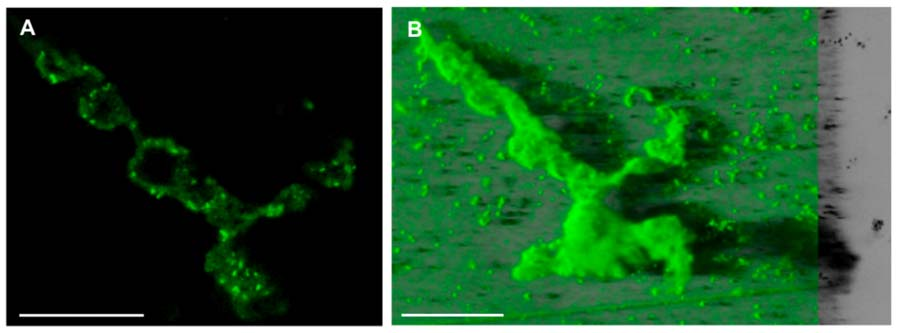
Cell lysis and formation of hollow voids inside microcolonies in the presence of As(III). 2D-confocal micrograph (**A**) and 3D-confocal reconstruction using IMARIS software (**B**) of a 72 h-old 2.67 mM As(III)-exposed biofilm structure. Cells were stained with SYTO9. Hollow voids appeared inside microcolonies subsequent to cell lysis. Scale bars: 50 µm.

This physiological heterogeneity was reinforced by cell lysis events which occurred inside arsenite exposed microcolonies after cell death events, generating hollow voids (Figure 3 and Figure S3).

**Figure 4:**
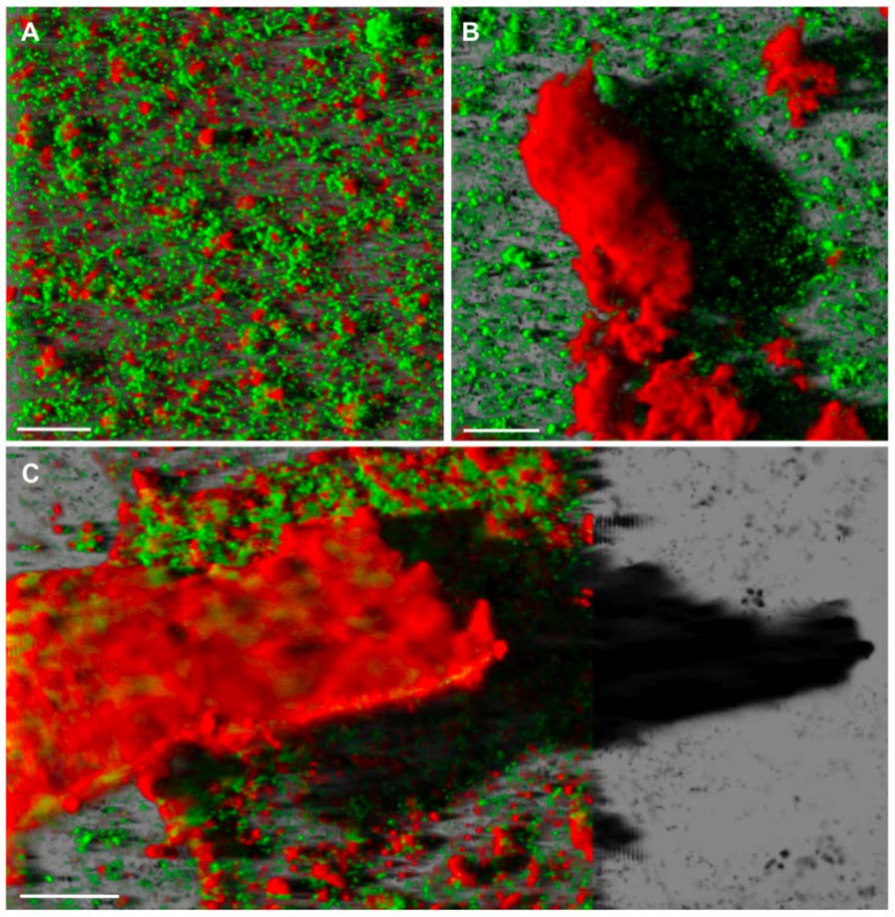
Three dimensional confocal reconstruction of 7 day-old biofilms. *Thiomonas* sp. CB2 biofilms were cultivated in the absence (**A**) or in the presence of 1.33 mM (**B**) and 2.67 mM As(III) (**C**). Biofilms were stained using SYTO9 (cells, green) and ConA (exopolysaccharide, red). Arsenic exposure induced altered biofilm architecture, leading to the formation of sponge- (**B**) and coral-like structures (**C**). Scale bars: 20 µm.

Concomitantly to cell lysis, the three-dimensional biofilm structures underwent complex rearrangements. This phenomenon began after 72 hours of culture (Figure 3) and increased with time. Indeed, after 7 days of culture, we observed tall three dimensional biofilm structures only in the presence of 1.33 and 2.67 mM As(III) (Figure 4). These structures, up to 90 µm high, were covered by large amounts of exopolysaccharides.

Cell lysis events are known to result in the liberation of nucleic acids. In order to determine the role of extracellular nucleic acids in *Thiomonas* sp. CB2 biofilm architecture, 72 h-old biofilms were treated with a nuclease. This treatment led to the disintegration of the biofilms grown in the presence of 1.33 mM As(III), removing all non-surface attached cells. However, nuclease treatment had no effects on non-As(III)-exposed biofilms (Figure S4 and data not shown). This result demonstrates an essential role of extracellular nucleic acids in Thiomonas sp. CB2 three-dimensional biofilm structure.

### Promotion of biofilm dispersal under arsenite exposure

**Figure 5:**
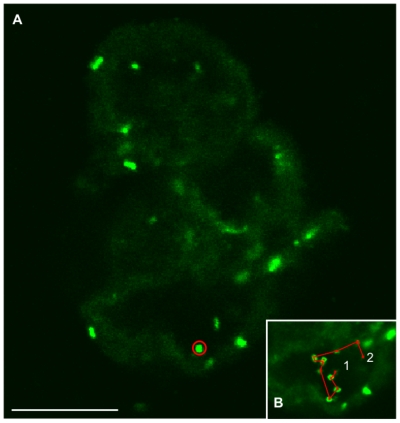
Motile bacteria swimming inside hollow voids. **A.** 2D-confocal representation of hollow voids of a 72 h-old 2.67 mM As(III)-exposed biofilm structure containing swimming bacteria. Cells were stained with SYTO9. Scale bar: 20 µm. **B.** 2D-time progression, in red, from point 1 to point 2 of the cell circled on panel (**A**). The representation is a projection of 16 confocal images taken at 1.63 second intervals.

Exploring the three-dimensional As(III)-exposed biofilm structures revealed the existence of subpopulations of highly motile cells inside the hollow biofilm voids generated by cell lysis events (Figure 5 and Movie S1). These remobilized bacteria were present in at least 72 h-old biofilms only in the presence of As(III) and promoted biofilm dispersal as they were observed to evacuate from the hollow voids of 7 days-old 2.67 mM As(III)-exposed biofilms (Movie S2).

In order to determine whether this bacterial As(III)-exposed biofilm population had acquired specific capacities regarding arsenic stress, we measured As(III) resistance of the cellular population originating from 72 h-old biofilm grown either in the absence of As(III) or in the presence of 1.33 mM As(III). Biofilm cells were plated on 10.7 mM As(III) containing solid medium, *i.e.* 0.7 mM more than the minimal inhibitory concentration initially determined for the strain. The formed colonies displayed the same convex and undulate morphology as those formed by the wild-type strain without As(III). The percentage of cells able to grow in the presence of 10.7 mM As(III) was of 0.2±0.05 and 19.9±7.28 (mean ± SD) for the population originating from biofilm grown in the absence and in the presence of As(III), respectively. The biofilm structural dynamics under As(III) exposure led also to the development of cells exhibiting an higher As(III) resistance than that observed for the initial strain.

## Discussion

Bacteria of the *Thiomonas* genus are ubiquitously found in arsenic contaminated sites, including AMD waters. We have recently shown that they are particularly well suited to survive in such extreme environments, combining several defence mechanisms, including arsenite metabolism and DNA repair. We have hypothesized that these strains, in particular *Thiomonas* sp. CB2, form biofilms in response to As(III) stress [11]. Indeed, during planktonic growth the CB2 strain formed flocs, *i.e.* bacterial aggregates, and this propensity was increased in the presence of As(III) but not in the presence of As(V). In the present study, the *Thiomonas* sp. CB2 biofilm development kinetics was compared in the absence and in the presence of As(III) at subinhibitory concentrations under static culture conditions. The overall biofilm development appeared to be strongly modified in response to As(III).

Under such conditions, the biofilm integrity may be reinforced by the high quantities of exopolysaccharide synthesised in the presence of As(III), these compounds being known to play an essential role in biofilm cohesion [4]. Even if the molecular determinants regulating this increased exopolysaccharide synthesis have not been precisely identified due to the current lack of mutagenesis tools for *Thiomonas* spp., four genes involved in dTDP-rhamnose biosynthesis, an exopolysaccharide precursor, may play a role in this process. Indeed, these genes are also present in closely-related strains, *i.e. Thiomonas* sp. 3As [11] and *Acidithiobacillus ferrooxidans* [18]. However, these genes were not found to be significantly over-expressed in *Thiomonas* sp. CB2 biofilm cells under As(III) exposure. This suggests that these genes are not the main genetic determinants involved in the exopolysaccharide synthesis induction highlighted by the ConA staining in the presence of As(III).

During biofilm formation, the presence of As(III) led to localized cell death and lysis events inside microcolonies. Such events have been previously described in other strains and several hypotheses have been proposed, including the coexistence of subpopulations of cells differing in their physiological state [19], [20]. In *Thiomonas* sp. CB2, a similar mechanism may explain the cell death events we observed inside the microcolonies of the arsenite-exposed biofilms. Indeed, arsenic-tolerance requires active cell physiology, *i.e.* the extensive synthesis of detoxification enzymes, efflux pumps, and DNA repair systems [21]. The cells located in the centre of the microcolonies, known to exhibit low metabolic activity [22], may also be more susceptible to this stress.

Localized cell death and lysis events are thought to contribute to the increased antibacterial compound tolerance in biofilm cells, in part because the lysis of a cell subpopulation provides nutrients and cellular polymers to protect the remaining cells [19], [23]. More importantly, these events appear to be of prime importance in the subsequent developmental processes we observed in the presence of arsenite in *Thiomonas* sp. CB2 biofilm. Indeed, extracellular nucleic acids were shown to be essential components of the biofilm matrix, playing a key structural role in this strain. Their liberation during cell lysis may also reinforce the structures and allow the subsequent biofilm developmental steps which led to the formation of the tall biofilm structures we observed only in the presence of As(III).

The three-dimensional development of the biofilm under As(III) exposure enhanced the biofilm heterogeneity and may also favour the development of distinct subpopulations of cells [22]. This hypothesis is supported by the presence of subpopulations of remobilized cells inside the hollow voids formed during the cell lysis events. These highly motile cells promoted the seeding dispersal of the strain by evacuating from the biofilm hollow voids. Such an active biofilm dispersal mechanism allows the strain to colonize new environments [24]. Moreover, about 20% of the bacterial As(III)-exposed biofilm population exhibited an As(III) resistance higher than that of the wild-type strain. Even if this increased resistance is probably mainly linked to the induction of enzymatic defence mechanisms [21], a part of this subpopulation of cells may also represent genetic variants, as described in other biofilms, *e.g.* those formed by *Pseudomonas aeruginosa* and *Streptococcus pneumoniae* [25], [26]. A high frequency of genomic rearrangement events inside *Thiomonas* spp. biofilms may also explain the high genomic diversity of the strains belonging the *Thiomonas* genus, which has been previously shown by comparative genomic analysis [11]. This hypothesis is currently under investigation.

In conclusion, we showed that the overall *Thiomonas* sp. CB2 biofilm development was markedly modified in response to subinhibitory arsenite concentrations leading to an increased As(III) resistance and, more importantly, to a rapid dispersal of part of the community. Our results suggest also that, in *Thiomonas* sp. CB2, the formation of a biofilm plays several roles in the arsenite resistance process. It is tempting to speculate that the survival and persistence of the strain in its extreme environment relies on a rapid biofilm development followed by the dispersal of a more resistant population. *Thiomonas* spp. may be persistent in AMD waters and thus assure a long-term role in the natural remediation process occurring in this site. More importantly, these processes may confer an important selective advantage by allowing the strain to survive in harsh conditions and also to colonize more favourable environments. These biofilm-associated processes presumably constituting an adaptive strategy may be widely conserved among bacteria in response to adverse conditions, including in pathogenic biofilms exposed to antimicrobials compounds.

## Supporting Information

Movie S1 **Remobilized bacteria in hollow biofilm voids.** Motile bacteria swimming inside hollow voids of a 72 h-old 2.67 mM As(III)-exposed biofilm. Cells were stained with SYTO9. This movie contains 32 confocal images taken at 1.63 second intervals. Scale bar: 10 µm.
(AVI)

Movie S2 **Biofilm dispersal.** Motile bacteria dispersing from hollow voids of a 7 days-old 2.67 mM As(III)-exposed biofilm. Cells were stained with SYTO9. This movie contains 37 confocal images taken at 1.63 second intervals. Scale bar: 10 µm.
(AVI)

Figure S1 ***Thiomonas*** **sp. CB2 planktonic growth.** *Thiomonas* sp. CB2 planktonic growth in the absence (grey) or in the presence of either 1.33 (white) or 2.67 mM As(III) (blue). Due to the strain propensity to form flocs during planktonic growth, including in the absence of As(III), growth parameters were assessed by measuring every 24 h during 4 days the optical density at 600 nm of the cultures. Cultures were performed in liquid medium in independent triplicates. The presence of As(III) did not significantly influence strain growth (*t*-tests, *P*>0.05). Error bars represent standard deviations.
(PPT)

Figure S2 **Microcolonies formation.** 2D-confocal micrograph of a microcolony formed after 48 hours of incubation in the absence of arsenic. Cells were stained with SYTO9 (green) and SYTOX Red (red). No dead cells, *i.e.* stained by SYTOX Red, were visible. Scale bar: 5 µm.
(PPT)

Figure S3 **Microcolony of a 72 h-old** ***Thiomonas*** **sp. CB2 biofilm unexposed to As(III).** 2D-confocal micrograph of a representative microcolony of a 72 h-old As(III)-unexposed biofilm. Cells were stained with SYTO9 (green) and SYTOX Red (red). No dead cells, *i.e.* stained by SYTOX Red, were visible. Unlike As(III)-exposed biofilms, microcolonies of biofilms unexposed to As(III) were filled with living cells. Scale bar: 10 µm.
(PPT)

Figure S4 **Nuclease treatment of the biofilm.** Role of extracellular nucleic acids in the biofilm structure. **a.** Three-dimensional confocal reconstruction of a representative 1.33 mM As(III)-exposed 72 h-old *Thiomonas* sp. CB2 biofilm using IMARIS software. Cells were stained with SYTO9 (green) and exopolysaccharides with ConA (red). **b.** Fluorescence microscope image of a 1.33 mM As(III)-exposed 72 h-old biofilm treated with Benzonase® Nuclease. All non-surface attached cells were removed by the treatment. Cells were stained with SYTO9 (green).
(PPT)

Table S1 **Primers used for quantitative RT-PCR.** List of primers used to perform the quantitative RT-PCR experiments.
(DOC)
