# Supplementary figures and images for: Subinhibitory Arsenite Concentrations Lead to Population Dispersal in Thiomonas sp
Source: PLoS One. 2011 Aug 18;6(8):e23181. doi: 10.1371/journal.pone.0023181 (PMC3158062; doi:10.1371/journal.pone.0023181)

## Slide 1
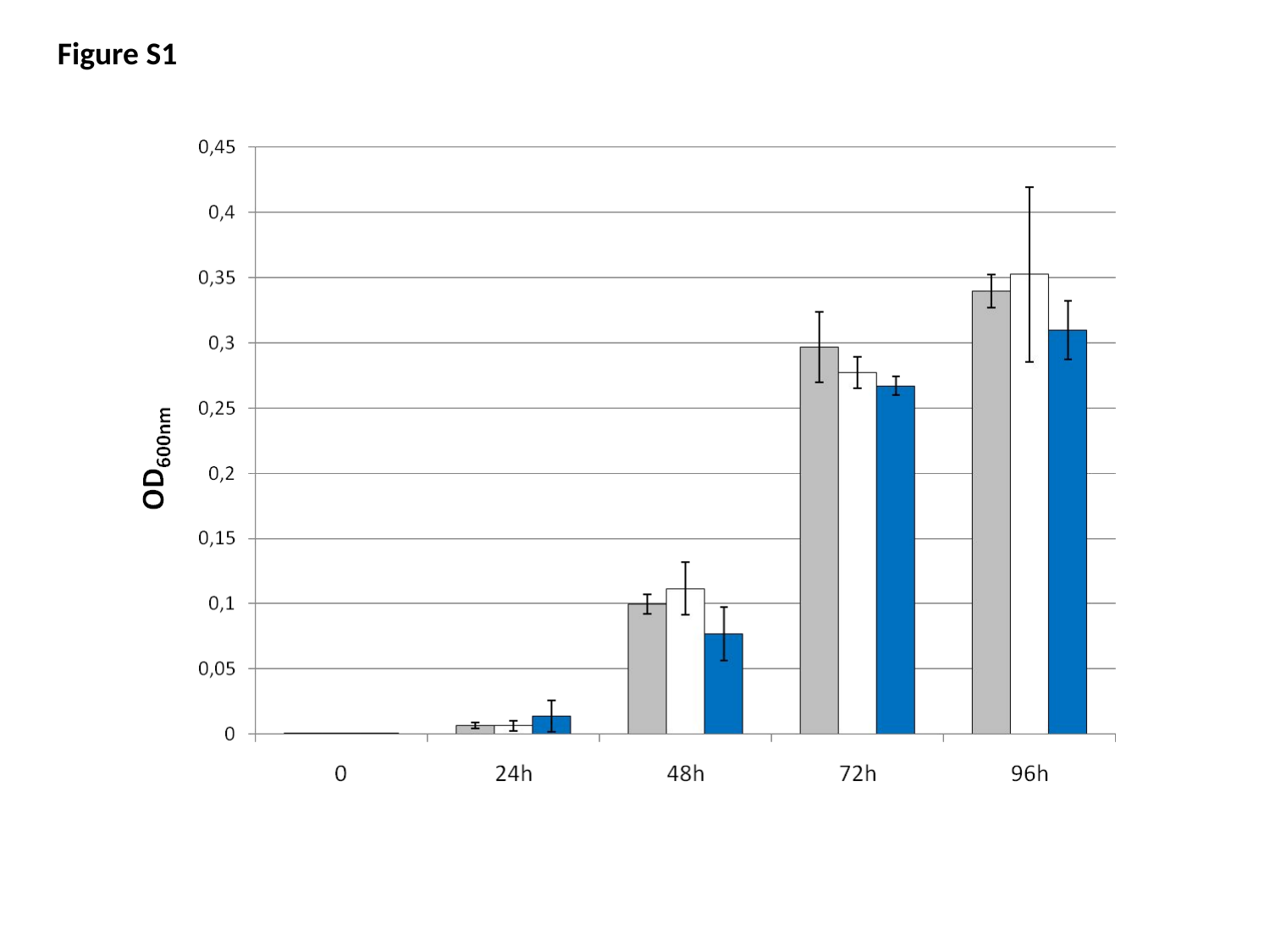

Figure S1

Supplement: Figure S1 — Thiomonas sp. CB2 planktonic growth. Thiomonas sp. CB2 planktonic growth in the absence (grey) or in the presence of either 1.33 (white) or 2.67 mM As(III) (blue). Due to the strain propensity to form flocs during planktonic growth, including in the absence of As(III), growth parameters were assessed by measuring every 24 h during 4 days the optical density at 600 nm of the cultures. Cultures were performed in liquid medium in independent triplicates. The presence of As(III) did not significantly influence strain growth (t-tests, P>0.05). Error bars represent standard deviations. (PPT) [file pone.0023181.s003.ppt]

## Slide 1
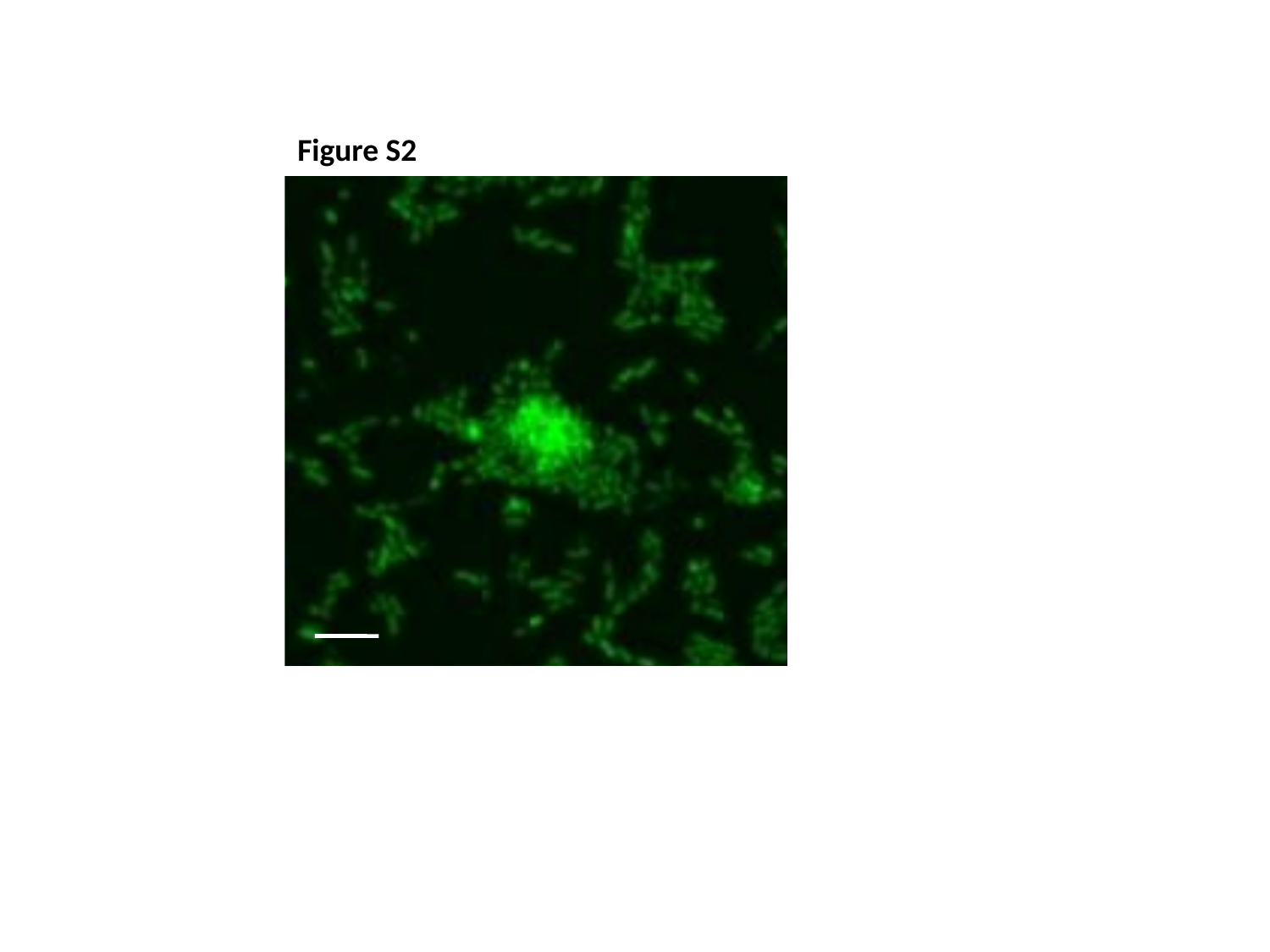

Figure S2

Supplement: Figure S2 — Microcolonies formation. 2D-confocal micrograph of a microcolony formed after 48 hours of incubation in the absence of arsenic. Cells were stained with SYTO9 (green) and SYTOX Red (red). No dead cells, i.e. stained by SYTOX Red, were visible. Scale bar: 5 µm. (PPT) [file pone.0023181.s004.ppt]

## Slide 1
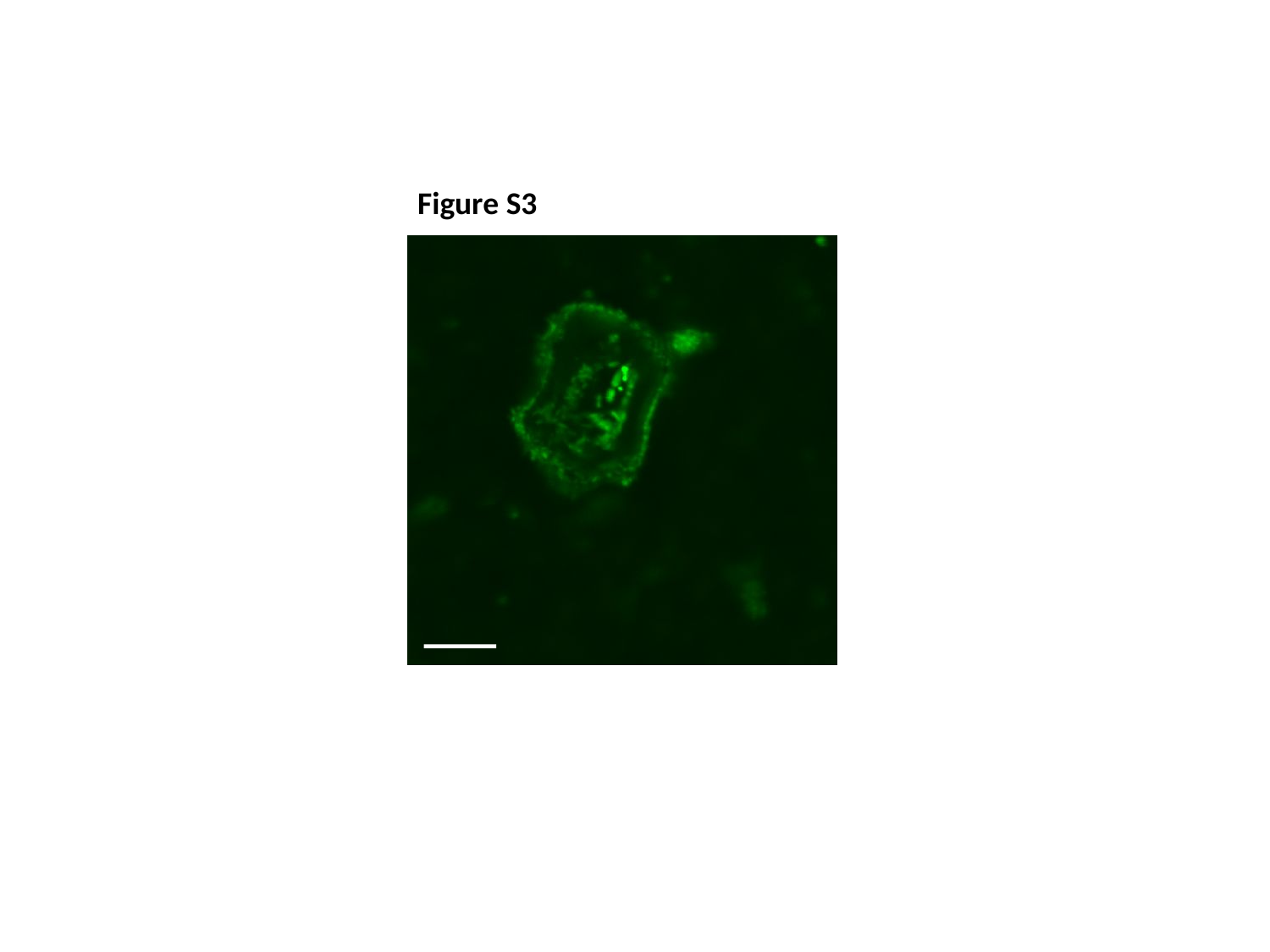

Figure S3

Supplement: Figure S3 — Microcolony of a 72 h-old Thiomonas sp. CB2 biofilm unexposed to As(III). 2D-confocal micrograph of a representative microcolony of a 72 h-old As(III)-unexposed biofilm. Cells were stained with SYTO9 (green) and SYTOX Red (red). No dead cells, i.e. stained by SYTOX Red, were visible. Unlike As(III)-exposed biofilms, microcolonies of biofilms unexposed to As(III) were filled with living cells. Scale bar: 10 µm. (PPT) [file pone.0023181.s005.ppt]

## Slide 1
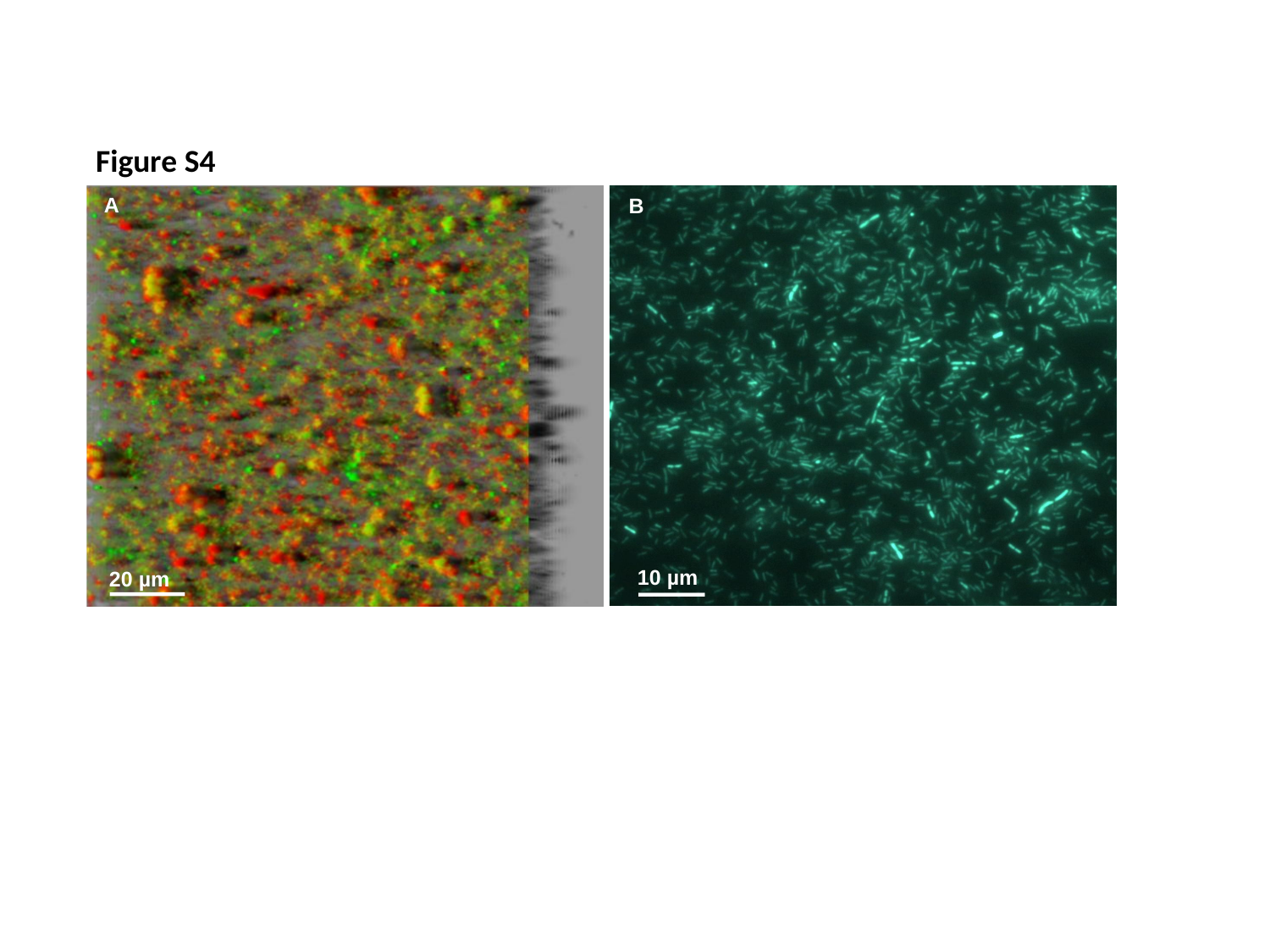

Figure S4
A
B
10 µm
20 µm

Supplement: Figure S4 — Nuclease treatment of the biofilm. Role of extracellular nucleic acids in the biofilm structure. a. Three-dimensional confocal reconstruction of a representative 1.33 mM As(III)-exposed 72 h-old Thiomonas sp. CB2 biofilm using IMARIS software. Cells were stained with SYTO9 (green) and exopolysaccharides with ConA (red). b. Fluorescence microscope image of a 1.33 mM As(III)-exposed 72 h-old biofilm treated with Benzonase® Nuclease. All non-surface attached cells were removed by the treatment. Cells were stained with SYTO9 (green). (PPT) [file pone.0023181.s006.ppt]
