# Supplementary material for: Subinhibitory Arsenite Concentrations Lead to Population Dispersal in Thiomonas sp
Source: PLoS One. 2011 Aug 18;6(8):e23181. doi: 10.1371/journal.pone.0023181 (PMC3158062; doi:10.1371/journal.pone.0023181)
Supplement: Table S1 — Primers used for quantitative RT-PCR. List of primers used to perform the quantitative RT-PCR experiments. (DOC) [file pone.0023181.s007.doc]

Table S1 “Primers used for quantitative RT-PCR”

| **Primer name** | **Primers sequence (5’-3’)** |
| --- | --- |
| **THI_2580 forward** | GGCGTCCGAGCGTTCGTAAAC |
| **THI_2580 reverse** | GCTGCATCGTCGATCGCTAC |
| **THI_0556 forward** | GGCACGGCGATATGCACATC |
| **THI_0556 reverse** | GCGGCAATTCGTCGAGCATC |
| **THI_1854 forward** | CGGTGGCTACGTGCAGAATC |
| **THI_1854 reverse** | GCAGCAGGCACAACGGAGTG |
| **THI_2969 forward** | GTGCCTGGCATCACACCTAC |
| **THI_2969 reverse** | CGGCAGTGGTCTTGCACATAC |
| **THI_2968 forward** | CGAACACGTAGTCGGTGGAG |
| **THI_2968 reverse** | CGAGCAGCCTGAGCTTATCG |
